# Supplementary material for: Cytokine Profile at Diagnosis Affecting Trough Concentration of Infliximab in Pediatric Crohn’s Disease
Source: Biomedicines. 2022 Sep 23;10(10):2372. doi: 10.3390/biomedicines10102372 (PMC9598182; doi:10.3390/biomedicines10102372)
Supplement: Supplementary file 1 [file biomedicines-10-02372-s001.zip › biomedicines-1848261-supplementary.pdf]

Supplementary Information

Supplementary Table S1. Evaluation of differences in cytokines according to disease severity and phenotype using Pearson correlation analysis.

		Hematocrit	Albumin	ESR	CRP	Calprotectin	PCDAI score	SES-CD score
**TNF-α, pg/mL**	**Pearson coefficient**	−0.197	−0.135	0.028	0.231	0.017	0.183	0.286
	**P value**	0.297	0.476	0.881	0.219	0.928	0.333	0.125
**Interleukin 6, pg/mL**	**Pearson coefficient**	−0.210	−0.026	0.033	0.019	0.019	0.099	0.388
	**P value**	0.265	0.891	0.861	0.548	0.922	0.604	**0.034**
**Interleukin 10, pg/mL**	**Pearson coefficient**	0.119	−0.048	0.004	0.167	0.063	0.100	−0.285
	**P value**	0.530	0.801	0.982	0.379	0.739	0.601	0.128
**Interleukin 17A, pg/mL**	**Pearson coefficient**	0.124	−0.081	0.015	0.161	0.046	0.252	0.217
	**P value**	0.513	0.670	0.395	0.394	0.809	0.180	0.250

TNF; Tumor necrosis factor, PCDAI; Pediatric Crohn's disease activity index, SDS-CD; Simple endoscopic score for Crohn's disease.

Supplementary Table S2. Comparison of initial cytokine profiles in patients treated with an anti-TNF-α who achieved endoscopic remission versus those who did not achieve endoscopic remission.

	**Infliximab use + ER (N = 18)**		**Infliximab use + No ER (N = 8)**		**P value**
	**Mean**	**SD**	**Mean**	**SD**	
**TNF-α, pg/mL**	16.2	12.0	21.8	14.1	0.405[a]
Interleukin 6, pg/mL	25.1	26.6	25.7	17.5	0.944[a]
Interleukin 10, pg/mL	110.8	76.0	137.2	134.2	0.636[a]
Interleukin 17A, pg/mL	9.3	6.8	11.5	9.6	0.592[a]

ER; Endoscopic remission, SD; Standard deviation, TNF; Tumor necrosis factor.

(a)

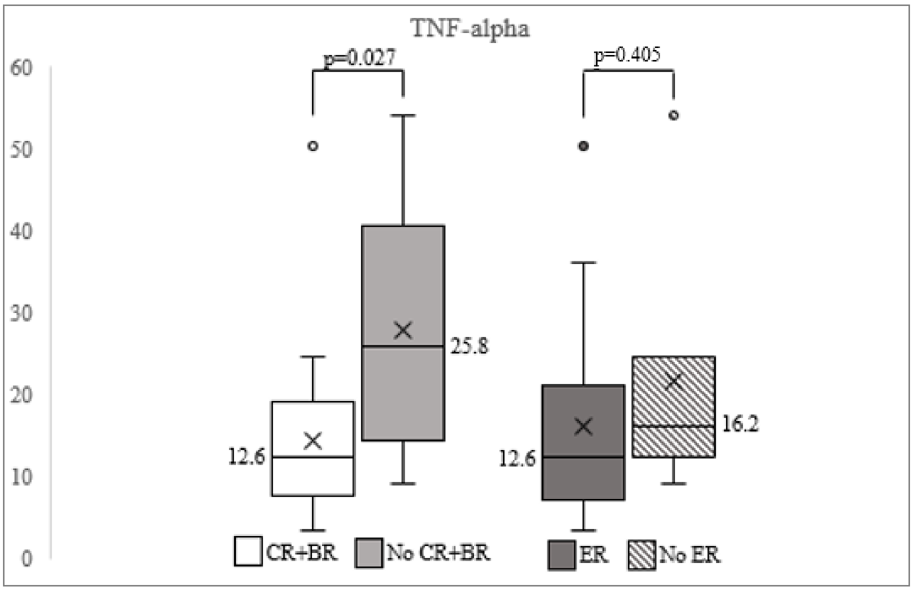

(b)

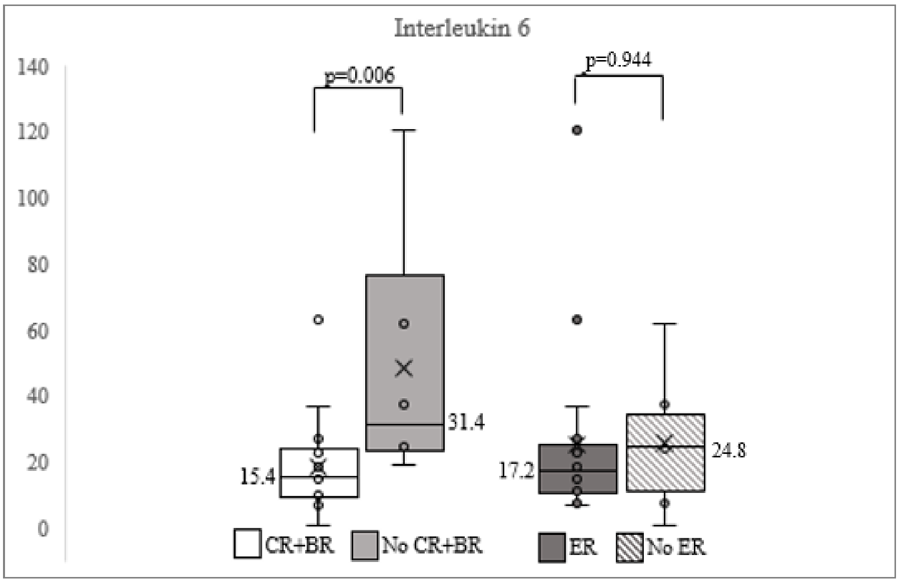

(c)

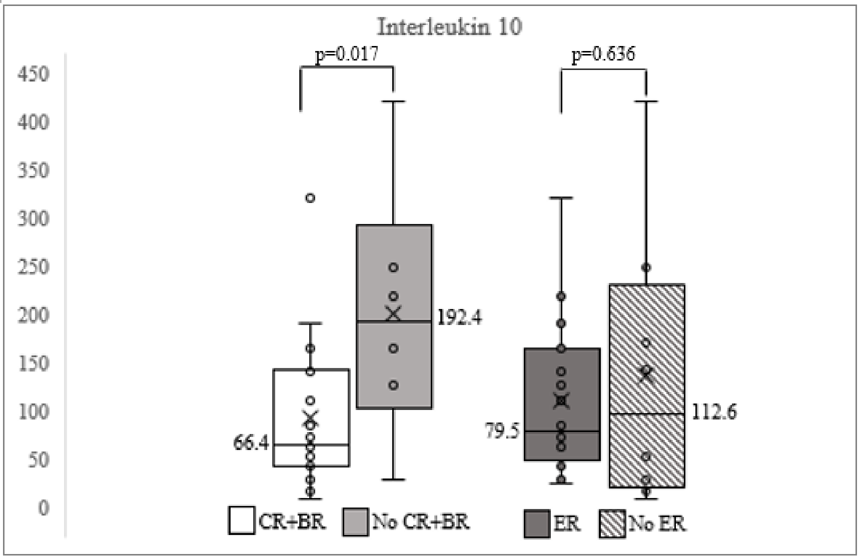

(d)

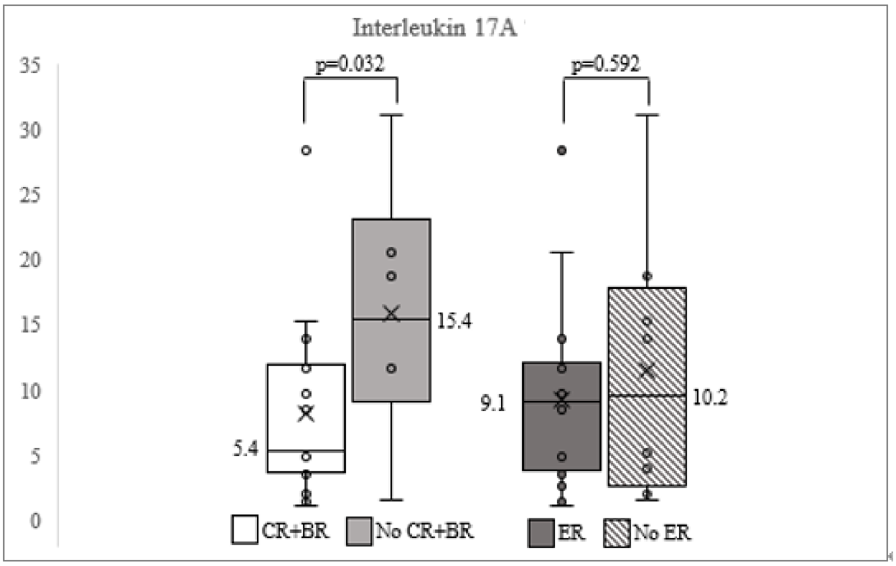

Supplementary Figure S1. Comparison of the (a) TNF-α, (b) Interleukin 6, (c) Interleukin 10, and (d) Interleukin 17A concentrations of patients with and without clinical and biochemical remission, and patients with and without endoscopic remission in patients receiving anti-TNF alpha treatment.
